# Unveiling Charge Carrier Recombination, Extraction, and Hot‐Carrier Dynamics in Indium Incorporated Highly Efficient and Stable Perovskite Solar Cells

**DOI:** 10.1002/advs.202103491

**Published:** 2022-02-13

**Authors:** Chaocheng Zhou, Tianju Zhang, Chao Zhang, Xiaolin Liu, Jun Wang, Jia Lin, Xianfeng Chen

**Affiliations:** ^1^ State Key Laboratory of Advanced Optical Communication Systems and Networks School of Physics and Astronomy Shanghai Jiao Tong University Shanghai 200240 China; ^2^ Department of Physics Shanghai Key Laboratory of Materials Protection and Advanced Materials in Electric Power Shanghai University of Electric Power Shanghai 200090 China; ^3^ Laboratory of Micro‐Nano Optoelectronic Materials and Devices, Shanghai Institute of Optics and Fine Mechanics Chinese Academy of Sciences Shanghai 201800 China; ^4^ Center of Materials Science and Optoelectronic Engineering University of Chinese Academy of Sciences Beijing 100049 China; ^5^ CAS Center for Excellence in Ultra‐intense Laser Science Shanghai 201800 China; ^6^ Collaborative Innovation Center of Light Manipulation and Applications Shandong Normal University Jinan 250358 China

**Keywords:** defect passivation, energy‐level alignment, exciton binding energy, hot‐carrier relaxation, perovskite solar cells

## Abstract

Perovskite solar cells (PSCs) have been propelled into the limelight over the past decade due to the rapid‐growing power conversion efficiency (PCE). However, the internal defects and the interfacial energy level mismatch are detrimental to the device performance and stability. In this study, it is demonstrated that a small amount of indium (In^3+^) ions in mixed cation and halide perovskites can effectively passivate the defects, improve the energy‐level alignment, and reduce the exciton binding energy. Additionally, it is confirmed that In^3+^ ions can significantly elevate the initial carrier temperature, slow down the hot‐carrier cooling rate, and reduce the heat loss before carrier extraction. The device with 1.5% of incorporated In^3+^ achieves a PCE of 22.4% with a negligible hysteresis, which is significantly higher than that of undoped PSCs (20.3%). In addition, the unencapsulated PSCs achieve long‐term stability, which retain 85% of the original PCE after 3,000 h of aging in dry air. The obtained results demonstrate and promote the development of practical, highly efficient, and stable hot‐carrier‐enhanced PSCs.

## Graphical abstract

In this article, state‐of‐the‐art perovskite solar cells were reported with a small amount of incorporated indium (In^3+^) ions. The In^3+^ ions accumulate at the buried interface and align the energy level to the electron transport layer. Additionally, the charge carrier dynamics at and above the band edge in mixed cation and halide perovskites can be greatly affected.

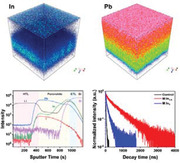

## 1 Introduction

Metal halide perovskite materials with the general structure of ABX_3_ (A = Cs^+^, CH_3_NH_3_^+^ (MA^+^, methylammonium), or CH(NH_2_)^2+^ (FA^+^, formamidinium); B = Pb^2+^ or Sn^2+^; X = Cl^−^, Br^−^, or I^−^) have garnered considerable attention for applications in light‐emitting devices,^[^ ^1^, ^2^, ^3^ ^]^ lasing,^[^ ^4^ ^]^ sensors,^[^ ^5^ ^]^ smart windows,^[^ ^6^ ^]^ photodetectors,^[^ ^7^ ^]^ and solar cells,^[^ ^8^, ^9^, ^10^, ^11^ ^]^ owing to their superior optoelectronic properties, such as appropriate band gap,^[^ ^12^, ^13^ ^]^ high optical absorption coefficient,^[^ ^14^ ^]^ long carrier lifetime,^[^ ^15^, ^16^ ^]^ long carrier diffusion length,^[^ ^17^, ^18^, ^19^ ^]^ and low exciton binding energy.^[^ ^20^, ^21^ ^]^ In particular, perovskite solar cells (PSCs) are considered as the most promising next‐generation photovoltaic (PV) devices owing to their low‐cost fabrication and outstanding performance. Over the past decade, the power conversion efficiency (PCE) of PSCs has rapidly increased from 3.8% to above 25%.^[^ ^22^, ^23^ ^]^ The main challenge in the future commercialization of PSCs is the further improvement of perovskite materials to achieve superior PCE and excellent stability under ambient environmental conditions. Different strategies have been explored to address these limitations; among these strategies, doping has been proven to be an effective method to influence the charge carrier dynamics, and enhance both the efficiency and long‐term stability of PSCs.^[^ ^24^ ^]^

Majority of the existing PV cells follow the assumption in the Shockley–Queisser thermodynamic detailed balance calculation; the photogenerated charge carriers cool down completely to the semiconductor band edges before extraction to the external load. This assumption results in a Shockley–Queisser limit PCE of 33% in single‐junction PV cells under 1‐sun AM1.5G illumination.^[^ ^25^ ^]^ It was previously proposed that if hot carriers can be collected before they lose their excess energy via relaxation processes, the theoretical maximum PCE of an ideal hot‐carrier solar cell can be further increased to 66%.^[^ ^26^, ^27^, ^28^, ^29^ ^]^ Slowing the cooling rate of hot carriers (less than 10 ps) before hot‐carrier extraction is a main challenge,^[^ ^30^ ^]^ particularly at low light excitation concentrations (i.e., comparable with photoexcitation under 1‐sun AM1.5G illumination). Studies have revealed that hybrid halide perovskites exhibit unusual slow cooling of hot carriers with above‐bandgap excitation compared with that of traditional semiconductors.^[^ ^31^ ^]^ Recently, it was reported that Zn^2+^ ions can create a new delocalized state at the R‐point of the Brillouin zone, which provides a channel for slowing down the hot electron relaxation in all‐inorganic halide perovskites.^[^ ^32^ ^]^ Therefore, the Zn‐doped thin film exhibits a hot‐carrier energy loss rate approximately three times smaller than that of the undoped film for hot carriers at 500 K and a low photoexcitation level of 10^17^ cm^−3^. Hence, the slowing down of hot‐carrier cooling and the efficient utilization of hot carriers in halide perovskite can significantly enhance the PSC performance.

Indium (In^3+^) has been previously introduced into all‐inorganic halide perovskites to reduce the defect density and retard crystallization.^[^ ^33^, ^34^ ^]^ In addition, Pb–In binary perovskite films exhibit high quality with multiple ordered crystal orientations, which are beneficial for efficient charge transport along multiple directions, leading to an improved PSC performance.^[^ ^35^ ^]^ However, there is still a lack of corresponding studies on the application of In^3+^ in high‐performance PSCs, and the underlying mechanism is still unclear. We incorporated a small amount of In^3+^ ions with Cs/MA/FA‐based highly efficient mixed cation and halide perovskites for developing Cs_0.05_(MA_0.17_FA_0.83_)_0.95_Pb(I_0.83_Br_0.17_)_3_:*x*In, which is abbreviated as M:In*_x_* (M and *x*% denote mixed perovskite and molar ratio of In^3+^:Pb^2+^, respectively). The In^3+^ ions can passivate the defects in the perovskite film, reduce the deep trap state, and lower the exciton binding energy. In addition, In^3+^‐incorporated perovskite substantially increased the initial hot‐carrier temperature and reduced the hot‐carrier energy loss rate at a low excitation density close to the 1‐sun illumination condition (carrier density ≈10^17^ cm^−3^). The M:In_1.5_ PSCs achieved a high PCE of 22.4% and retained 85% of the original PCE after 3000 h of exposure to dry air.

## 2 Results and Discussion

### 2.1 Stable In^3+^‐Incorporated Perovskite Thin Films

**Figure 1:**
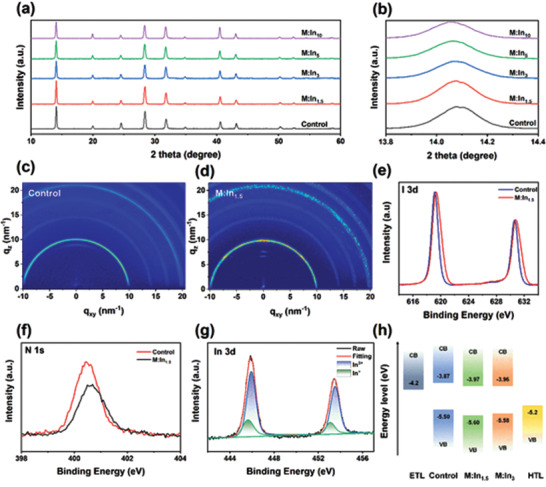
XRD and XPS characterizations of M:In*_x_* perovskite thin films. a,b) XRD patterns of M:In*_x_* films with *x* = 0, 1.5, 3, 5, and 10. GIWAXS patterns of c) control and d) M:In_1.5_ films. XPS data for e) I 3d, f) N 1s, and g) In 3d core levels of control and M:In_1.5_ perovskite films. h) Derived energy band diagram of the control, M:In_1.5_, and M:In_3_ perovskite films.

The crystalline phase and quality of perovskite thin films are essential for PSC performance. **Figure** **1**a shows the X‐ray diffraction (XRD) patterns for M:In*_x_* with *x* = 0, 1.5, 3, 5, and 10. All compositions showed typical perovskite peaks at ≈14.1° and 28.4°, which corresponded to the (110) and (220) lattice planes, respectively. After In^3+^ doping, no other peaks appeared, indicating that the incorporation of In^3+^ ions did not form any other phases. For undoped thin films, we observed a tiny peak of cubic PbI_2_ (001) plane at ≈12.6°; however, this peak disappeared when In^3+^ was incorporated, indicating the full conversion of PbI_2_ to perovskite (Figure S1, Supporting Information). Furthermore, with an increase in the In^3+^ ratio, the intensity of the peaks at 31.8° and 40.6°, corresponding respectively to the (310) and (224) lattice planes increased. This suggests that In^3+^ ions induced perovskite growth with ordered crystal orientations.^[^ ^35^ ^]^ The ionic radius of In^3+^ is 69 pm, which is smaller than Pb^2+^ (119 pm). The smaller radius allows the In^3+^ ions interstitial doping or partial replacement of Pb^2+^. If the In^3+^ ions partially replace the Pb^2+^, the perovskite lattice will shrink. However, the diffraction peaks of XRD patterns gradually shifted to lower angles with further In^3+^ incorporation, as shown in Figure 1b. The calculated interplanar crystal spacings of the (110) plane of five M:In*_x_* films with *x* = 0, 1.5, 3, 5, and 10 are 6.287, 6.291, 6.292, 6.298, and 6.300 Å, respectively. This result indicates that In^3+^ ions were inserted into the interstices of the perovskite lattice and filled the Pb vacancy defects on the surface which enlarged the lattice. Furthermore, the M:In_1.5_ perovskite exhibited the smallest full width at half maximum (FWHM) of the (110) diffraction peak, implying the largest average crystallite size of the M:In_1.5_ film (Figure S2, Supporting Information). 2D grazing‐incidence wide‐angle X‐ray scattering (GIWAXS) was utilized to probe more structure information (Figure 1c,d). The M:In_1.5_ film showed relatively strong spots in the ring patterns, and the azimuthally integrated scattering intensity of different GIWAXS patterns along the ring at scattering vector *q* = 10 nm^−1^ which represents the (110) plane of the corresponding perovskite lattice was plotted in Figure S3 (Supporting Information). The integrated intensity of M:In_1.5_ is larger than the control sample and an obvious peak at the azimuth angle of 85° appears, implying the preferentially oriented crystal growth in the M:In_1.5_ film. The morphologies of the thin films were investigated using scanning electron microscopy (SEM). As shown in Figure S4 (Supporting Information), the M:In_1.5_ film has a larger average grain size than that of the control film, resulting in a lower trap state density at the surface and grain boundaries in the In^3+^‐incorporated thin films. However, as the ratio of In^3+^ further increased to 3%, the grain size decreased, consistent with the XRD results. For In^3+^ ratios above 5%, pinholes appeared in the thin films (Figure S4d, Supporting Information); these pinholes deteriorated the PSC performance. The energy‐dispersive X‐ray spectroscopy (EDS) elemental mapping images confirmed that the In element was uniformly distributed on the thin film surface (Figure S5, Supporting Information). Time of flight secondary ion mass spectrometry (ToF‐SIMS) was performed to examine the vertical ion distribution. The longitudinal distribution of Pb and I decreases slightly with increasing depth and is relatively uniform overall, whereas In^3+^ ions accumulate at the bottom of the perovskite film (Figures S6 and S7, Supporting Information), indicating that the In^3+^ ions prefer to passivate the defects at the buried surface rather than in the bulk.

To verify the incorporation of In^3+^, we performed high‐resolution X‐ray photoelectron spectroscopy (XPS) of the perovskite films. Figure 1e shows that the binding energy of the I 3d of In^3+^‐incorporated perovskite thin films exhibited two main peaks located at 630.8 and 619.4 eV, whereas that of the control film showed peaks at 630.6 and 619.1 eV, corresponding to I 3d_3/2_ and I 3d_5/2_, respectively. The peaks of the I 3d orbital are slightly shifted toward higher binding energies with In^3+^ doping, which might be attributed to the higher energy of the In–I bond. In addition, the N 1s orbital is also shifted to a higher binding energy with In^3+^ incorporation, as shown in Figure 1f. This can reduce halide vacancy defects and stabilize the organic cations.^[^ ^36^ ^]^ The results were further confirmed using Raman spectroscopy (see Figure S8 in the Supporting Information). The mode near 282 cm^−1^ is ascribed to the out‐of‐plane FA^+^ bending, which showed an increased intensity with In^3+^, implying that In^3+^ ions prevented organic cation decomposition. We also noticed the decreased intensity when the ratios of In^3+^ increased to 5%, which is ascribed to the poor film quality of M:In_5_ sample. As shown in Figure S4 (Supporting Information), the morphology of M:In_5_ exhibits severe pinholes. The spectrum of the In 3d orbital was fitted by In^+^ (445.6 and 453.1 eV) and In^3+^ (445.9 and 453.5 eV) components, as shown in Figure 1g. The presence of In^+^ indicates the formation of In^+^–In^3+^ redox shuttle, where In^3+^ oxidized Pb defects (In^3+^ + Pb → Pb^2+^ + In^+^), and In^+^ reduced I defects (In^+^ + 2I → In^3+^ + 2I^−^) in a cyclical transition. This phenomenon is comparable to the Eu^3+^–Eu^2+^ doping systems, as In^3+^–In^+^ ion pairs have a similar redox potential as that of Eu^3+^–Eu^2+^ pairs.^[^ ^37^ ^]^

To investigate the distribution of energy levels, we employed UV–visible (UV–Vis) absorption spectroscopy and ultraviolet photoelectron spectroscopy (UPS). Upon increasing the amount of In^3+^, we found that the absorption edge of the absorption spectra was slightly red‐shifted. The bandgaps of the control, M:In_1.5_, and M:In_3_ perovskites determined by the Tauc plot were ≈1.631, 1.628, and 1.615 eV, respectively (Figure S9, Supporting Information). Furthermore, UPS was employed to assess the energy level shift. The Fermi level (*E*_F_) was evaluated using the first derivative of cutoff region as −3.64 (control), −4.15 (M:In_1.5_), and −4.05 eV (M:In_3_) (Figure S10, Supporting Information). The valence band maximum (VBM) was calculated as follows

(1) $$E_{F}-\;E_{V}=\;E-h\upsilon \;\left(h\upsilon \;=\;21.22\;\mathrm{eV}\right)$$

where (*E*_F_ −  *E*_V_) is the distance from VBM to the Fermi level, and *E* is evaluated using the VB edge region as 19.34 (control), 19.75 (M:In_1.5_), and 19.67 eV(M:In_3_). Thus, the VBM values are estimated to be −5.50 (control), −5.60 (M:In_1.5_), and −5.58 eV (M:In_3_). The conduction band minimum (CBM) is the sum of the VBM and bandgaps. The CBM and VBM of M:In_1.5_, which are 0.1 eV lower than that of the control, improved the energy‐level alignment, as illustrated in Figure 1h. This was beneficial for extracting electrons.

### 2.2 Charge Carrier Recombination and Extraction Characteristics

**Figure 2:**
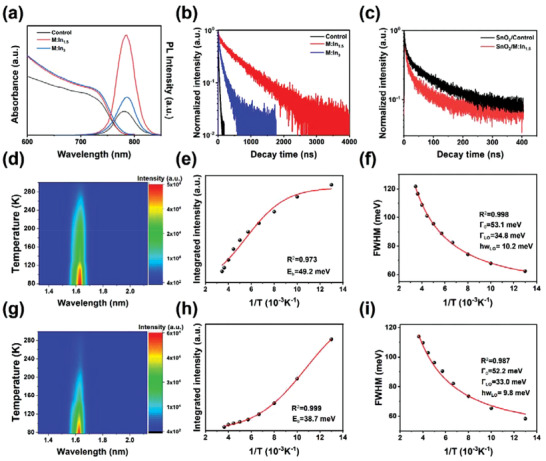
Optical characterizations of the M:In*_x_* perovskite films. a) UV–Vis absorption and PL spectra. TRPL of b) glass/perovskite and c) glass/SnO_2_/perovskite. The contour plot of temperature‐dependent PL spectra of the d) control and g) M:In_1.5_ thin films. Integrated PL intensity and FWHM of e,f) the control and h,i) M:In_1.5_ thin films as a function of temperature, respectively.

The photoluminescence (PL) spectra of the perovskite films deposited on the glass substrates are shown in **Figure** **2**a. The PL peaks of the control, M:In_1.5_, and M:In_3_ samples are located at 780, 783, and 786 nm, respectively, showing the same red shift as that of the absorption spectra with In^3+^ incorporation. All the thin films exhibited a small Stokes shift of ≈20 nm. In addition, the M:In_1.5_ film exhibited the strongest PL intensity with the smallest PL FWHM of 38.2 nm, implying suppressed nonradiative recombination and weakened electron–phonon scattering intensity.^[^ ^17^ ^]^

Time‐resolved PL (TRPL) spectra were measured to evaluate the interband charge carrier recombination dynamics in the M:In*_x_* thin films deposited on the glass substrates (Figure 2b). The decay curves were fitted using the biexponential equation as follows

(2) $$I\;\left(t\right)=A_{1}\exp\left(-\frac{t}{\tau_{1}}\right)+\;A_{2}\exp\left(-\frac{t}{\tau_{2}}\right)$$

where *τ*_1_ and *τ*_2_ represent the fast and slow decay processes, respectively. The former is related to the quenching of electrons and holes by trap states and interfacial charge transfer, whereas the latter is related to the radiative recombination of the electrons and holes.^[^ ^38^, ^39^ ^]^ The PL decay lifetime of the M:In_1.5_ film increased significantly compared with that of the control perovskite film, with *τ*_1_ increasing from 4.76 to 54.0 ns and *τ*_2_ increasing from 24.3 to 736 ns; this result implies that the incorporation of In^3+^ ions reduces the trap state density and suppresses nonradiative recombination. Consequently, M:In_1.5_‐based PSCs are prone to have high open‐circuit voltages owing to low nonradiative energy loss, which is promising for highly efficient PSCs. More detailed parameters of the carrier lifetime are shown in Table S1 (Supporting Information). When perovskite films were deposited on a SnO_2_ electron transport layer (ETL), the decay lifetimes (both *τ*_1_ and *τ*_2_) of the M:In_1.5_ film were shorter than that of the control sample (for the control film *τ*_1_ = 6.78 ns and *τ*_2_ = 96.28 ns; for M:In_1.5_ *τ*_1_ = 6.05 ns and *τ*_2_ = 64.67 ns), as shown in Figure 2c, indicating the faster charge extraction and reduced charge recombination. The superior interfacial charge transfer between ETL and M:In_1.5_ film is due to that the CBM of M:In_1.5_ is 0.1 eV lower than that of control, which leads to the more aligned energy level to ETL. To investigate the interface between perovskite and HTL, the TRPL of the device with the structure of glass/perovskite/Spiro‐OMeTAD was carried out (see Figure S11 in the Supporting Information). According to Figure 1h, the VBM of M:In_1.5_ is more mismatched to hole transport layer (HTL) than control film, which would lead to a longer PL lifetime of the device. However, the *τ*_1_ of control (15.4 ns) and M:In_1.5_ (15.3 ns) are similar, while the *τ*_2_ of the M:In_1.5_ film decreased from 93.4 to 65.85 ns. This unexpected result may be due to the In^3+^ ions preferring to accumulate at the buried surface of the perovskite film away from the interface between perovskite and HTL, which eliminates the energy barrier between the HTL and M:In_1.5_ perovskite film. Additionally, the small amount of In^3+^ passivates the surface defects, which suppresses the nonradiative recombination at the perovskite and HTL interface.

The temperature‐dependent PL spectra of the control and M:In_1.5_ films were measured to further explore the intrinsic photophysical mechanism of electron–hole pairs produced by light excitation and electron–phonon interaction in the temperature range of 77–300 K (Figure 2d,g). As the temperature decreased from 300 to 77 K, the PL intensity of both the control and M:In_1.5_ samples increased owing to the suppressed phonon‐assisted relaxation, and the PL peak position red‐shifted gradually owing to the narrowing of the bandgap. This behavior is apparently opposite to that of a typical semiconductor and is attributed to thermal contraction, which increases the overlap between the Pb 6s and I 5s antibonding atomic orbitals forming the VBM and reduces the overall band gap.^[^ ^40^ ^]^ The exciton binding energy represents the strength of the interaction between an electron and a hole, which is a crucial factor that influences the performance of optoelectronic devices by influencing carrier recombination. The radiative recombination rate is proportional to the binding energy; with a low binding energy, the excitons can be easily dissociated into free carriers and extracted. The PL integrated intensity as a function of temperature is depicted in Figure 2e,h, which can be fitted using the Arrhenius equation^[^ ^41^ ^]^

(3) $$I\;\left(T\right)=\;\frac{I_{0}}{1+Ae^{E_{b}/\left(k_{b}T\right)}}$$

where *I*(*T*) and *I*_0_ are the integrated PL intensities at *T* and 0 K, respectively; *E*_b_ is the exciton binding energy; and *k*_B_ is the Boltzmann constant. With 1.5% In^3+^ incorporation, the binding energy decreased from 49.2 to 38.7 meV, implying that In^3+^ suppressed the radiative recombination and promoted the carrier extraction. Furthermore, the FWHM of the PL peaks of both the control and M:In_1.5_ samples significantly increased as the temperature increased owing to the enhanced electron–phonon interaction (Figure 2f,i). The electron–phonon coupling strength can be extracted as follows^[^ ^42^ ^]^

(4) $$\Gamma \left(T\right)=\;\Gamma \left(0\right)+\;\sigma T+\;\frac{\Gamma_{\mathrm{LO}}}{e^{\hbar \omega_{\mathrm{LO}}/k_{B}T}-1}$$

where Γ(*T*) is the FWHM at temperature *T*; Γ(0) is the inhomogeneous broadening; *σ* is the interaction between electrons and acoustic phonons, which is negligible and set to 0 for fitting; ℏ*ω*_LO_ is the longitudinal optical (LO) phonon energy; and Γ_LO_ represents the electron–phonon coupling strength. The optical phonon energies of both the control and M:In_1.5_ samples are ≈10 meV, which is consistent with previous results.^[^ ^43^ ^]^ The values of Γ_LO_ of the control and M:In_1.5_ films are 34.8 and 33.0 meV, respectively, implying that the In^3+^ ions slowed down the rate of electron–phonon scattering, promoting electron extraction.

### 2.3 Hot Carrier Dynamics within the Perovskite Films

**Figure 3:**
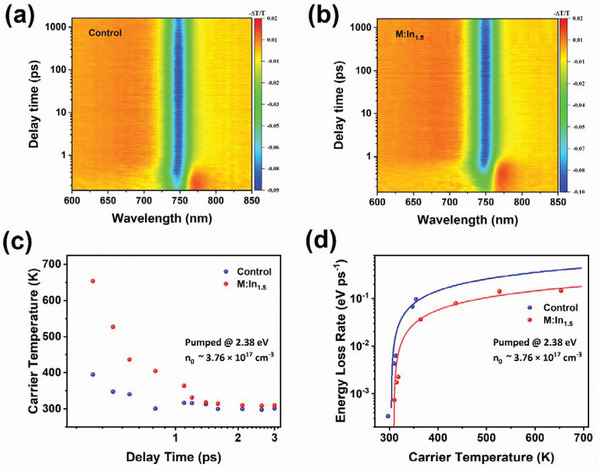
TA characterizations and hot‐carrier dynamics. a,b) TA measurements of the control and M:In_1.5_ perovskite films under excitation at 2.38 eV with a carrier density of 3.76 × 10^17^ cm^−3^. c) Average hot‐carrier temperature as a function of delay time, and d) energy loss rate as a function of carrier temperature of the control and M:In_1.5_ perovskite films at a pump photon energy of 2.38 eV and carrier densities of 3.76 × 10^17^ cm^−3^.

Transient absorption (TA) spectroscopy was utilized to further investigate the photophysical processes of the nonequilibrium interactions of photogenerated carriers. The In^3+^‐induced effect on hot‐carrier dynamics close to the 1‐sun illumination condition was investigated. **Figure** **3**a,b shows the false‐color 2D TA mapping of the M:ln_1.5_ and control thin‐film samples after excitation at 520 nm (2.38 eV) and a low carrier density of ≈3.76 × 10^17^ cm^−3^, respectively. The broadened ground‐state bleach band (GSB, negative signal) centered at ≈750 nm is caused by the band‐filling effect at the absorption band edge of the linear absorption spectrum.^[^ ^16^ ^]^ Moreover, in the range below the GSB peak (760−800 nm), photoinduced absorption (PIA, positive signal) appears. With an increase in the relaxation time, the GSB peak is red‐shifted, and the positive PIA signal is gradually transformed into a negative bleaching signal (Figure S12a, Supporting Information). In addition, the tail of the GSB peak from 700 to 720 nm, caused by a population of hot carriers, changes from a negative bleaching signal to a positive absorption (PA) signal.^[^ ^44^ ^]^ Global analysis indicates that the M:In_1.5_ film shows approximately twice longer carrier thermalization and cooling time as shown in Figure S12c,d (Supporting Information) (detailed analysis is presented in Supporting Information). The cooling dynamics and extraction of hot carriers at the interface are highly important for using the excess energy of hot carriers. Hence, we extracted the effective carrier temperature *T*_c_ to describe the quasi‐equilibrium distribution of the hot carriers. The high energy bleach tail above the bandgap can be approximately described by the Maxwell–Boltzmann distribution;^[^ ^45^ ^]^ thus *T*_c_ can be obtained by fitting the high‐energy bleach tail with following equation

(5) $$\frac{\Delta T}{T}\;\left(\hbar \omega \right)=A_{0}\;\left(\hbar \omega \right)\exp\left(-\frac{\hbar \omega }{k_{B}T_{c}}\right)$$

where ℏ*ω* is the probe energy; *A*_0_ is the linear absorbance. To ensure hot‐carrier scattering after the initial excitation, the hot‐carrier temperature was extracted after carrier thermalization at a time delay of 300 fs to 3 ps (Specific extraction process see Figure S13 in the Supporting Information). Figure 3c shows the time‐dependent carrier temperature of the control and M:In_1.5_ perovskite films at a low pump energy (2.38 eV) and carrier density (*n*_0_ ≈ 3.76 × 10^17^ cm^−3^). The initial carrier temperature of the control film at 300 fs was ≈395 K, whereas that of the M:In_1.5_ film was ≈650 K. Furthermore, the energy loss rate per carrier (*J*_r_) was calculated from the extracted hot‐carrier temperature using *J*_r_ = −1.5*k*_B_d*T*_c_/d*t*. As shown in Figure 3d, the energy loss rate decreased gradually from 0.15 to 0.14 eV ps^−1^ until *T*_c_ reached ≈500 K. Subsequently, *J*_r_ decreased rapidly as the hot‐carrier temperature approached the lattice temperature, owing to the carrier–phonon interaction process. This process can be fitted using the LO‐phonon interaction model (see Note S1 in the Supporting Information). Furthermore, for a hot carrier at 400 K, the energy loss rate of the M:In_1.5_ film (0.067 eV ps^−1^) is approximately three times smaller than that of the control film (0.182 eV ps^−1^). This slow energy loss rate is promising for utilizing the excess hot‐carrier energy, which might be helpful for enhancing the open‐circuit voltage in M:In_1.5_‐based devices.^[^ ^45^, ^46^, ^47^, ^48^ ^]^

### 2.4 PV Performance of PSCs

**Figure 4:**
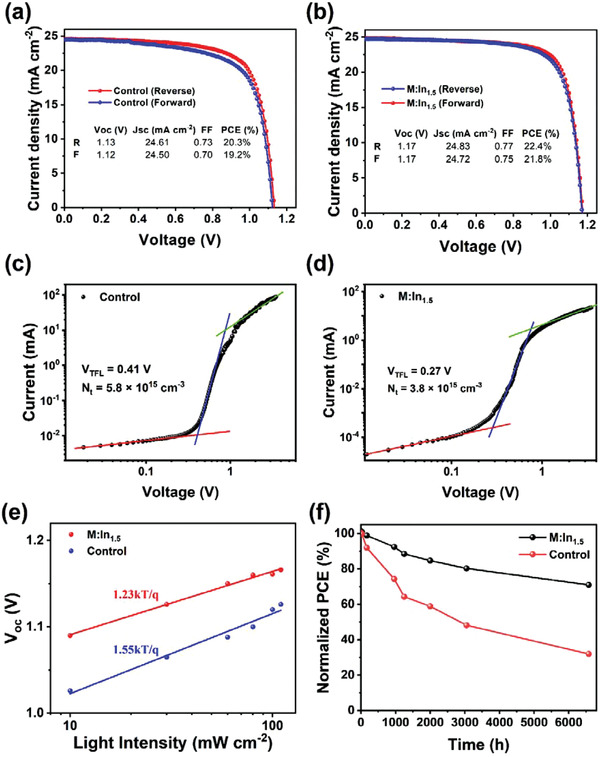
Device characterizations. a,b) *J*–*V* curves of the control and M:In_1.5_ devices. c,d) SCLC of the electron‐only devices. e) Dependence of *V*_OC_ with light intensity of the control and M:In_1.5_ devices. f) Stability of the control and M:In_1.5_ devices.

To investigate the PV performance, we fabricated PSCs with the structure of FTO/SnO_2_/perovskite/spiro‐OMeTAD/Au. The cross‐sectional SEM image indicated the formation of vertically oriented grains (Figure S14, Supporting Information). The photocurrent density–voltage (*J*–*V*) curves were obtained under 1‐sun AM1.5G illumination with a light intensity of 100 mW cm^−2^, as shown in **Figure** **4**a,b. The control PSCs showed a short‐circuit current density (*J*_SC_) of 24.60 mA cm^−2^, open‐circuit voltage (*V*_OC_) of 1.13 V, and fill factor (FF) of 0.73, leading to a PCE of 20.3% in the reverse scan; furthermore, they exhibited a *J*_SC_ of 24.50 mA cm^−2^, *V*_OC_ of 1.12 V, and FF of 0.70, leading to a PCE of 19.2% in the forward scan. After 1.5% In^3+^ incorporation, the *J*_SC_, *V*_OC_, and FF values of the PSCs were improved to 24.83 mA cm^−2^, 1.17 V, and 0.77, respectively, achieving a PCE of 22.4% in reverse scan; furthermore, the values in forward scan were 24.72 mA cm^−2^, 1.17 V, and 0.75, achieving a PCE of 21.8%. The hysteresis can be evaluated by the hysteresis index (HI) as follows

(6) $$\mathrm{HI}\;=\;\frac{\mathrm{PC}E_{\mathrm{reverse}}-\mathrm{PC}E_{\mathrm{forward}}}{\mathrm{PC}E_{\mathrm{reverse}}}$$

The HI values of the control and M:In_1.5_ devices were 0.054 and 0.026, respectively. Ion migration has been proposed as one of the basis for the hysteresis. We performed time of flight secondary ion mass spectrometry (ToF‐SIMS) to examine the ion distribution in the device before and after light irradiation aged test. The I and Pb ions of control device show an obvious migration after 30 min of light irradiation, while the M:In_1.5_ device exhibits negligible ion migration (see Figure S15 in the Supporting Information). This result implies that the In^3+^ can suppress the ion migration to reduce the hysteresis. With higher In^3+^ incorporation, the PCE gradually decreased. The best PCE of the M:In_3_ and M:In_5_ based devices were 21.0% and 19.2%, respectively. The detailed PV parameters of the devices modified with different concentrations of In^3+^ were summarized in Table S2 (Supporting Information). The corresponding external quantum efficiency (EQE) measurements (Figure S16, Supporting Information) showed that the integrated *J*_SC_ increased from 23.2 to 24.0 mA cm^−2^, owing to the wider range of absorption of the In^3+^‐doped sample, which was consistent with the absorption spectra. The main improvement appeared in *V*_OC_ and *FF*, where the *V*_OC_ increased from 1.13 to 1.17 V and FF increased from 0.73 to 0.77. The improvement in the *V*_OC_ of In^3+^‐based perovskite devices is mainly owing to the lower radiative and nonradiative recombination rates and extracted excess hot‐carrier energy above the perovskite bandgap.^[^ ^31^ ^]^ The superior interfacial contact and band alignment improved the FF of the In^3+^‐based devices. Compared to *V*_OC_, the *J*_SC_ slightly increased from 24.50 to 24.83 mA cm^−2^. This result indicates that the In^3+^ ions prefer to passivate the interface defects rather than bulk defects, which is consistent with the ToF‐SIMS result.

The space charge limited current (SCLC) was utilized to quantitatively examine the trap state density (*N*_t_) of the perovskite films. The electron‐only devices were fabricated with the structure of FTO/SnO_2_/perovskite/PCBM/Au. The dark *J*–*V* curves were divided into three parts by two kinks (Figure 4c,d). In the ohmic region, the current density increased gradually and linearly as the bias voltage increased. When the bias increased to the first kink, the trap states were filled with injected charges, and current density increased rapidly. The kink point is called trap‐filled limit voltage (*V*_TFL_), which is proportional to the trap state density and can be calculated as follows

(7) $$N_{t}=\frac{2\varepsilon \varepsilon_{0}V_{\mathrm{TFL}}}{eL^{2}}$$

where *ε* is the relative dielectric constant; *ε*_0_ is the vacuum permittivity; *e* is the elementary charge; and *L* is the thickness of the perovskite layer. The value of *V*_TFL_ of the electron‐only device is 0.41 and 0.27 V for the control and M:In_1.5_ samples, respectively. The calculated trap density of M:In_1.5_ is 3.8 × 10^15^ cm^−3^, which is lower than that of the control device (5.8 × 10^15^ cm^−3^). This is because In^3+^ suppressed the Pb and halide vacancy defects and assisted thin‐film crystallization, which is beneficial to the improvement of *V*_OC_ and FF.^[^ ^49^ ^]^ The ideality factor (*n*) for the diode can be used to determine the dominant carrier recombination mechanism of the PSCs. Figure 4e shows that we can fit the dependence of *V*_OC_ on the light illumination intensity using the following equation

(8) $$n\;=\;\frac{e}{k_{B}T}\frac{dV_{\mathrm{oc}}}{\mathrm{dln}I}$$

The value of *n* for the control and M:In_1.5_ PSCs is 1.87 and 1.23, respectively, indicating that the defect‐assisted carrier recombination is effectively reduced after In^3+^ incorporation. This finding is consistent with the PL and TRPL results.

The stability of the PSCs stored in dry air (<10% RH) was further explored, as shown in Figure 4f. The In^3+^‐incorporated PSC retained over 88.5% of the original PCE after 1200 h, 85% after 3000 h, and 71% after 6500 h, while the control PSC retained only 32% of its original value after 6500 h. The results indicate that In^3+^ doping significantly improved the stability of the PSCs. To further confirm the stability enhancement, we collected the XRD patterns of the perovskite films after storage for 10 d in air at 30%–40% RH without encapsulation, as shown in Figure S17 (Supporting Information). The degradation rate of the perovskite films can be characterized by the separated amount of PbI_2_. After 10 days, the control film decomposed largely with an obvious PbI_2_ signal at 12.7°, whereas the In^3+^‐based perovskite film did not exhibit an apparent PbI_2_ peak, demonstrating that the In^3+^ ions considerably suppressed the perovskite film degradation. The XPS has also been performed to verify the air stability. After 25 d of exposure to air at about 40% RH without encapsulation, the XPS spectrum of the M:In_1.5_ perovskite film changed negligibly, whereas the control film degraded and appeared obvious metallic Pb^0^ signals as shown in Figure S18 (Supporting Information). This is attributed to the formation of the In^3+^–In^+^ redox shuttle which oxidized Pb^0^ to prevent the degradation of perovskite.

## 3 Conclusion

We demonstrated that the slight incorporation of In^3+^ ions can greatly affect the charge carrier dynamics at and above the band edge in mixed cation and halide perovskites. The PL and temperature‐dependent PL spectra indicated that In^3+^ ions effectively inhibited radiative and nonradiative recombination and increased the carrier lifetime, with *τ*_1_ from 4.76 to 54.0 ns and *τ*_2_ from 24.3 to 736 ns. This is due to the In^3+^‐assisted perovskite crystallization and filling of the Pb and halide vacancies. The CBM of In^3+^ doped film was 0.1 eV lower than that of pristine perovskite film, which aligned energy level to ETL. And the In^3+^ ions preferred to accumulate at the buried surface away from the interface between perovskite and HTL, which eliminated the energy barrier between the HTL and In^3+^ doped perovskite film. The initial hot‐carrier temperature of the In^3+^ ion‐incorporated perovskite film was doubled. In addition, the energy loss rate for the hot carrier at ≈400 K was one‐third that of the control film at a low excitation density close to the 1‐sun illumination condition (≈10^17^ cm^−3^). This leads to a longer time required for intraband hot carrier cooling, which is beneficial for utilizing hot carriers. Therefore, the best In^3+^‐based perovskite device achieved a significantly improved PCE of 22.4% with a negligible hysteresis. In addition, the In^3+^‐doped PSCs showed excellent stability in dry air, which could maintain 85% of the initial PCE after 3000 h of aging. The obtained results demonstrate and promote the development of practical, highly efficient, and stable PSCs.

## 4 Experimental Section

### Materials

PbI_2_ (99.999%, Sigma‐Aldrich), PbBr_2_ (99%, Sigma‐Aldrich), CsI (99.9%, Sigma‐Aldrich), methylammonium bromide (MABr, 99%, Deysol), formamidinium iodide (FAI, 99%, Deysol), 2′,7,7′‐tetrakis‐(*N*,*N*‐di‐*p*‐methoxyphenylamine)‐9,9′‐spirobifluorene (Spiro‐MeOTAD, 99.5%, Xi'an Polymer Light Technology Corp.), 4‐tert‐butylpyridine (tBP, 96%, Xi'an Polymer Light Technology Corp.), lithium bis(trifluoromethanesulfonyl)imide (Li‐TFSI, 99%, Xi'an Polymer Light Technology Corp.), SnO_2_ colloid precursor (15% in H_2_O, Alfa Aesar), chlorobenzene (CB, 99.8%, Sigma‐Aldrich), acetonitrile (99.9%, Sigma‐Aldrich), *N*,*N*‐dimethylformamide (DMF, 99.8%, Sigma‐Aldrich), dimethyl sulfoxide (DMSO, 99.8%, Sigma‐Aldrich), and fluorine‐doped tin oxide (FTO)‐coated glass substrates (Shanghai Materwin Corp.) were used as received.

### Device Fabrication

FTO glass substrate was cleaned sequentially in detergent, deionized water, ethanol, and isopropanol. Then the substrate was treated with oxygen plasma for 10 min and dried with an argon gun. The SnO_2_ layer was prepared by spin‐coating the SnO_2_ colloid precursor solution at 4000 rpm for 30 s and annealing at 150 °C for 30 min. To prepare FAPbI_3_ (MAPbBr_3_) perovskite precursor solution, the PbI_2_ (PbBr_2_) were dissolved in DMF:DMSO = 4:1 (v:v), and the solution was heated to 180 °C on a hot plate for about 10 min. After cooling to room temperature, the FAI (MABr) powder was added with the stoichiometry of FAI/PbI_2_ (MABr/PbBr_2_) = 1:1.09 (9% excess Pb). The MAFA perovskite precursor solution was obtained by mixing FAPbI_3_ and MAPbBr_3_ solutions with a volume ratio of 5:1. The CsMAFA perovskite precursor solution was prepared by adding 5% vol 1.5 m CsI stock solution to the MAFA precursor solution. The In^3+^‐incorporated perovskite precursor solution was prepared by adding 1.5% vol 1.5 m InI_3_. To deposit the perovskite film, the precursor solution was spin‐coated on FTO glass substrate in two steps, 10 s at 1000 rpm and then 20 s at 6000 rpm. 100 µL CB was dripped as the antisolvent on the perovskite film 5 s before ending. The HTL was prepared by spin‐coating Spiro‐OMeTAD (90 mg) in CB (1 mL) with tBP (39.5 µL), Li‐TFSI (23 µL, 520 mg mL^−1^ in acetonitrile), and FK209 (10 µL, 375 mg mL^−1^ in acetonitrile) additives at 4000 rpm for 10 s. Finally, 80 nm gold was thermally evaporated under vacuum as the top electrode.

### Characterizations

The X‐ray diffraction (XRD) patterns were recorded by X‐ray diffractometer (Bruker D8 Advance). The morphology of samples was acquired with scanning electron microscopy (SEM, Navigator‐100 and JSM‐7800F). The X‐ray photoelectron spectroscopy (XPS) measurement was carried out by Nexsa (Thermo Fisher). The ultraviolet photoelectron spectroscopy (UPS) was measured using ESCALAB 250Xi (Thermo Fisher). The UV–visible (UV–Vis) absorption spectra were measured using a SHIMADZU UV‐3600 spectrophotometer. The steady‐state photoluminescence (PL) and time‐resolved PL (TRPL) spectra were collected using a Zolix OmniFluo900 spectrofluorometer.

The *J*–*V* curves were measured with a source meter (Keithley 2400) and a solar simulator (Oriel Sol 3A, Newport) under AM1.5G (100 mW cm^−2^), which was calibrated with a standard silicon solar cell certificated by NREL. The *J*–*V* curves were measured in reverse (1.2 to −0.2 V) or forward (−0.2 to 1.2 V) scanning modes with 0.01 V step and 0.1 s time delay (scanning speed, 100 mV s^−1^). A black mask with an aperture area of 0.0785 cm^2^ was put on the surface of devices during measurements. The EQE spectra were obtained by an EQE measurement system (Newport).

Femtosecond transient absorption spectroscopy (fs‐TAS) measurements of perovskite films prepared on quartz substrates were performed using our home‐built TAS setup. The frequency doubled 520 nm output from a Spectra‐Physics Spirit laser (350 fs, 1 kHz, 40 µJ pulse^−1^) was used for pump beam, while a fraction was used for white light continuous (WLC) spectrum generation using a sapphire crystal. The pump beam was chopped at 500 Hz, and the WLC probe signals were collected using an ultrafast fiber optic spectrometer. The time window of TA measurement is 1.6 ns. Second, the TA spectra with 400 nm pump light was measured by HARPIA TA device, and the pump light was obtained employing Yb:KGW femtosecond laser spectroscopic system (Light Conversion Ltd.).

## Conflict of Interest

The authors declare no conflict of interest.

## Supporting information

Supporting Information

## Data Availability

The data that support the findings of this study are available from the corresponding author upon reasonable request.
