# Supplementary material for: Unveiling Charge Carrier Recombination, Extraction, and Hot‐Carrier Dynamics in Indium Incorporated Highly Efficient and Stable Perovskite Solar Cells
Source: Adv Sci (Weinh). 2022 Feb 13;9(11):2103491. doi: 10.1002/advs.202103491 (PMC9008790; doi:10.1002/advs.202103491)
Supplement: Supplementary file 1 — Supporting Information [file ADVS-9-2103491-s001.pdf]

ADVANCED SCIENCE
Open Access

Supporting Information

for *Adv. Sci.*, DOI 10.1002/advs.202103491

Unveiling Charge Carrier Recombination, Extraction, and Hot-Carrier Dynamics in Indium Incorporated Highly Efficient and Stable Perovskite Solar Cells

Chaocheng Zhou, Tianju Zhang, Chao Zhang, Xiaolin Liu, Jun Wang, Jia Lin and Xianfeng Chen**

ADVANCED SCIENCE
Open Access

Supporting Information

for *Adv. Sci.*, DOI: 10.1002/advs.202103491

Unveiling charge carrier recombination, extraction, and hot-carrier dynamics in indium incorporated highly-efficient and stable perovskite solar cells

Chaocheng Zhou, Tianju Zhang, Chao Zhang, Xiaolin Liu, Jun Wang, Jia Lin, and Xianfeng Chen**

Supporting Information

Unveiling charge carrier recombination, extraction, and hot-carrier dynamics in indium incorporated highly-efficient and stable perovskite solar cells

Chaocheng Zhou, Tianju Zhang, Chao Zhang, Xiaolin Liu, Jun Wang, Jia Lin, and Xianfeng Chen**

Note S1

The density of photogenerated carriers was calculated from the following formula:

$$n_0 = \frac{\mathrm{F}\alpha}{\mathrm{Lh}\nu}\varphi \qquad (1)$$

where F is the fluence of pump, α is the absorption coefficient of the material, L is the thickness of the sample, hv is the photon energy of the pump pulse, and φ is the ratio of free charge carrier produced per photon absorbed, which is generally assumed to be unity.

Note S2

The energy loss rate per carrier (J_r) can be calculated from the extracted hot carrier temperature with $J_r = -1.5k_B dT_c/dt$. At a low carrier density, hot-carrier cooling occurs primarily through carrier-LO phonon interaction. The hot carriers lose excess energy owing to the dominant Fröhlich interaction via LO phonon emission[1,2]. The LO phonon mode decays into the daughter acoustic phonon modes via phonon–phonon interaction before the hot-carrier temperature approaches the lattice temperature. Owing to the slow thermal equilibration between the LO phonon and acoustic phonons, the energy loss rate reduced rapidly when the hot-carrier temperature became equal to the lattice temperature. And the process can be fitted by the LO-phonon interaction model[3]:

$$J_r = \frac{3}{2}\frac{\hbar\omega_0}{\tau_{LO}}\left(e^{\frac{\hbar\omega_0}{kT_a}} - e^{\frac{\hbar\omega_0}{kT_c}}\right)\frac{N_{LO}(T_a)}{N_{LO}(T_a)}\frac{kT_c}{\hbar\omega_0}e^{\frac{\hbar\omega_0}{kT_c}} \qquad (2)$$

where $\hbar\omega_0$ is the phonon energy which is determined by temperature-dependent PL, $N_{LO}(T)$ is the LO phonon occupation number at temperature T, τ_{LO} is the characteristic LO-phonon lifetime, and T_a is the acoustic phonon temperature.

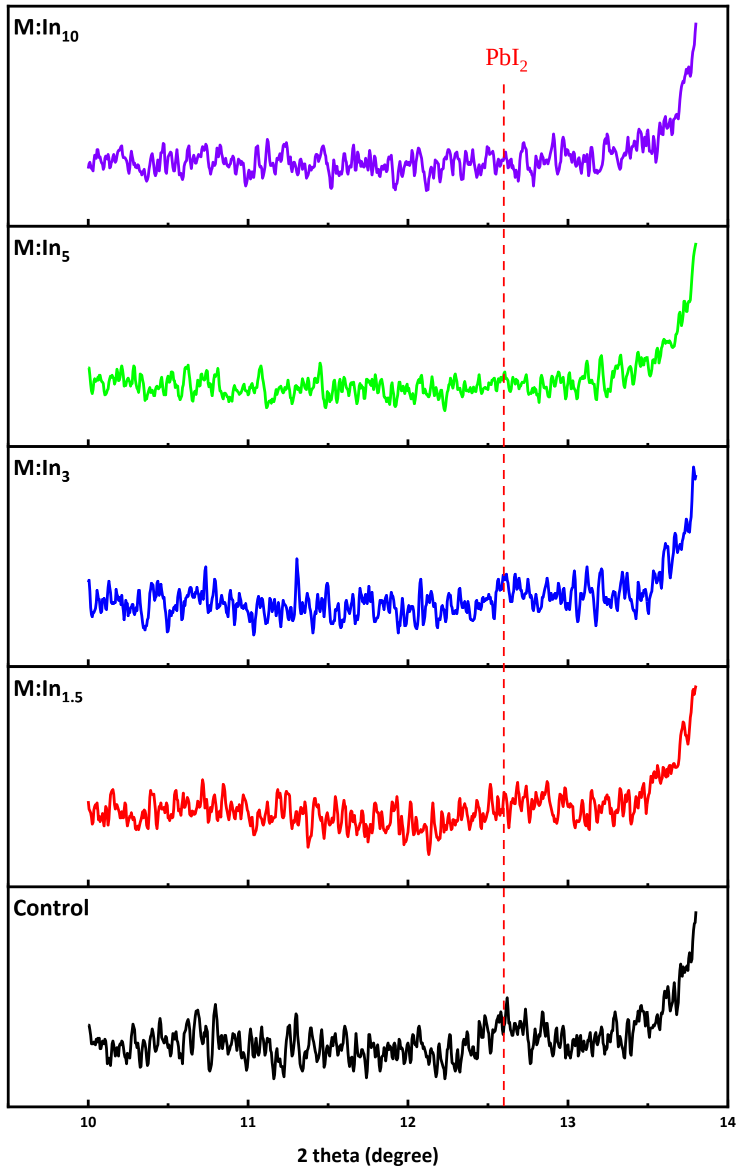

Supplementary Figure 1. XRD patterns of different types of perovskite films.

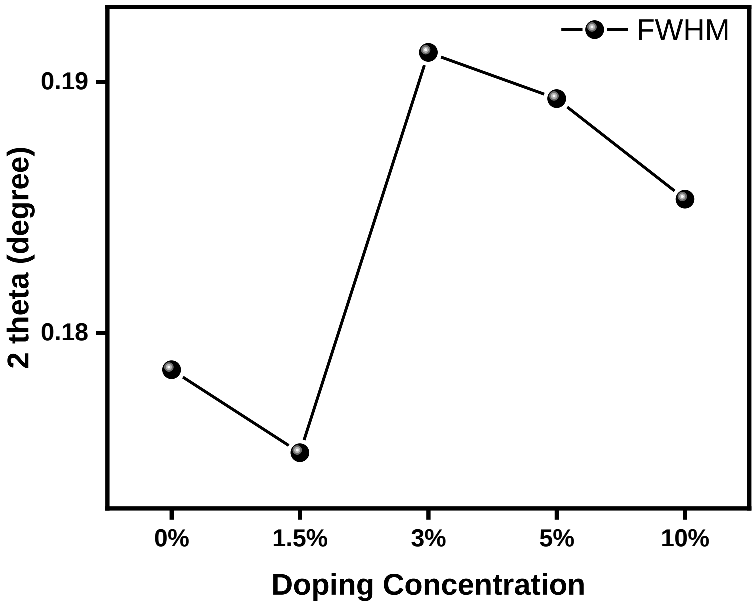

Supplementary Figure 2. FWHM of peak at about 14.1° of M:In$_x$ films with x = 0, 1.5, 3, 5, and 10.

The M:In$_{1.5}$ perovskite showed smaller FWHM than the control one, but when the In^{3+} ratio increased to above 3%, the FWHM enlarged significantly, indicating that the slight amount of In^{3+} can induce the better crystallization. The crystal size was calculated by Scherrer equation, $D = K\gamma/B\cos\theta$, where K is the Scherrer constant, γ is the wavelength of X-ray, B is the FWHM of the diffraction peak, and θ is the diffraction angle. The results showed that the crystal sizes of M:In$_x$ (x = 0, 1.5, 3, 5, and 10) were 116.7, 118.9, 109.0, 110.0, and 112.4 nm, respectively. Because there is no subtraction of the broadening of the instrument itself, the actual difference of the crystal size would be larger.

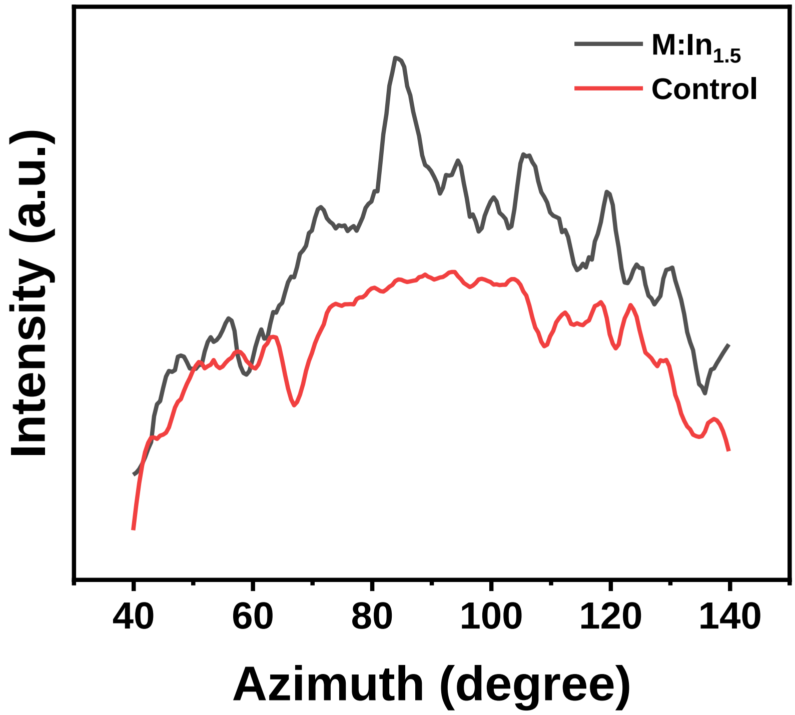

Supplementary Figure 3. Radially integrated intensity plots along the ring at scattering vector q = 10 nm^{-1} (110 plane) of different perovskite films.

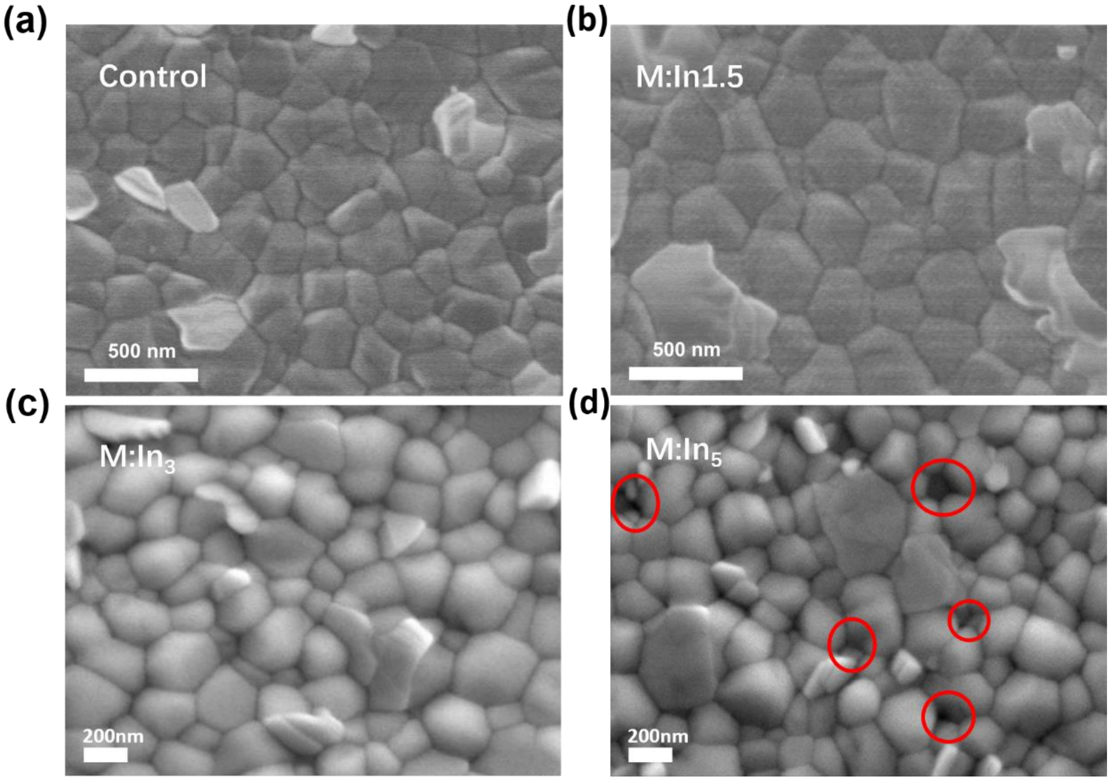

Supplementary Figure 4. Top-view SEM images of (a) control, (b) $M{:}In_{1.5}$, (a) $M{:}In_3$, and (b) $M{:}In_5$ thin films.

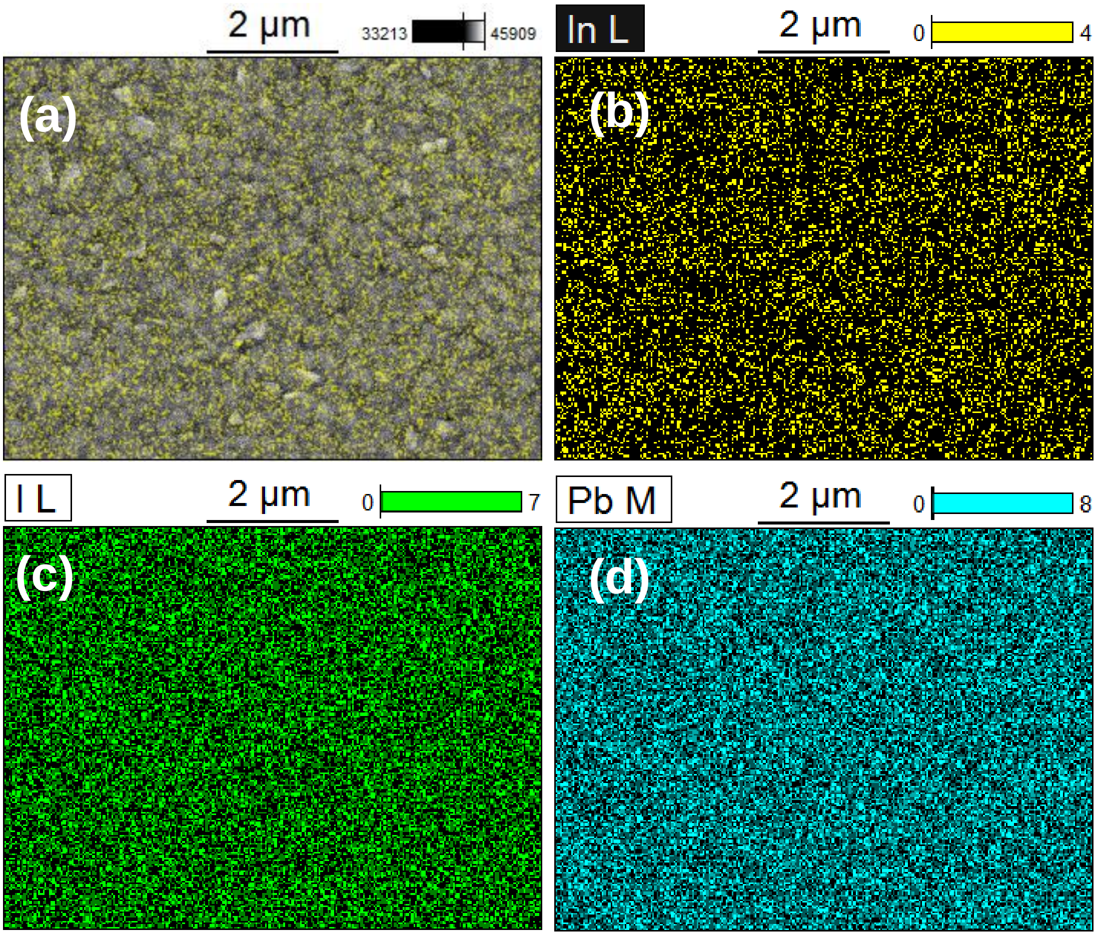

Supplementary Figure 5. (a) SEM image of M:$In_{1.5}$ perovskite film. EDS elemental mapping images of M:$In_{1.5}$ perovskite film: (b) In, (c) I, and (d) Pb.

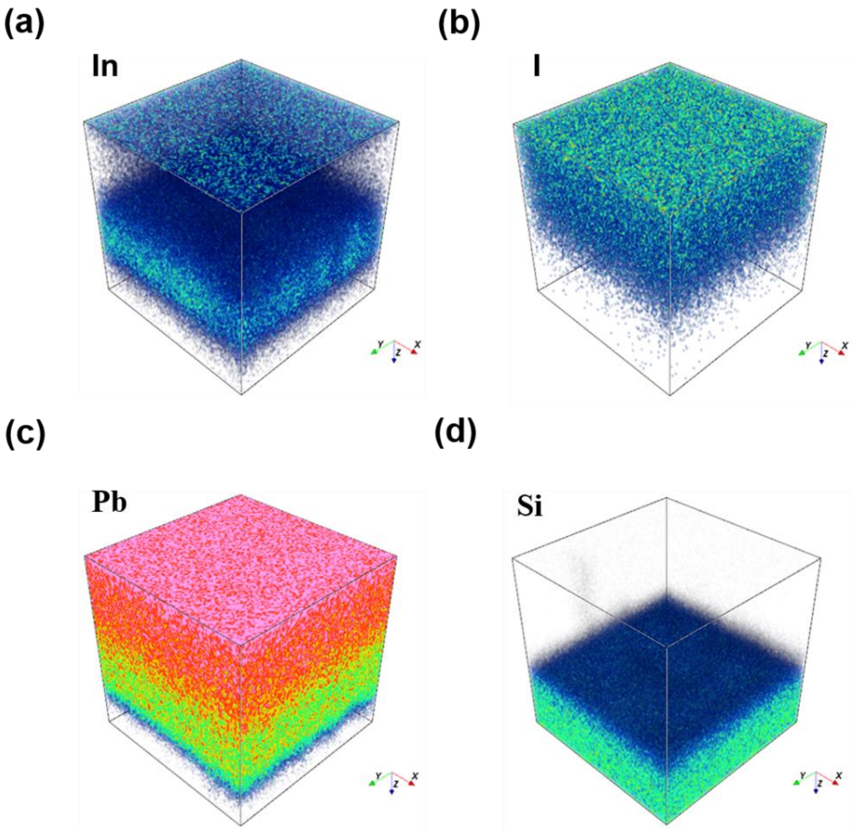

Supplementary Figure 6. 3D render overlay images of the control and M:$In_{1.5}$ perovskite films deposited on Si substrate with (a) In, (b) I, (c) Pb, and (d) Si.

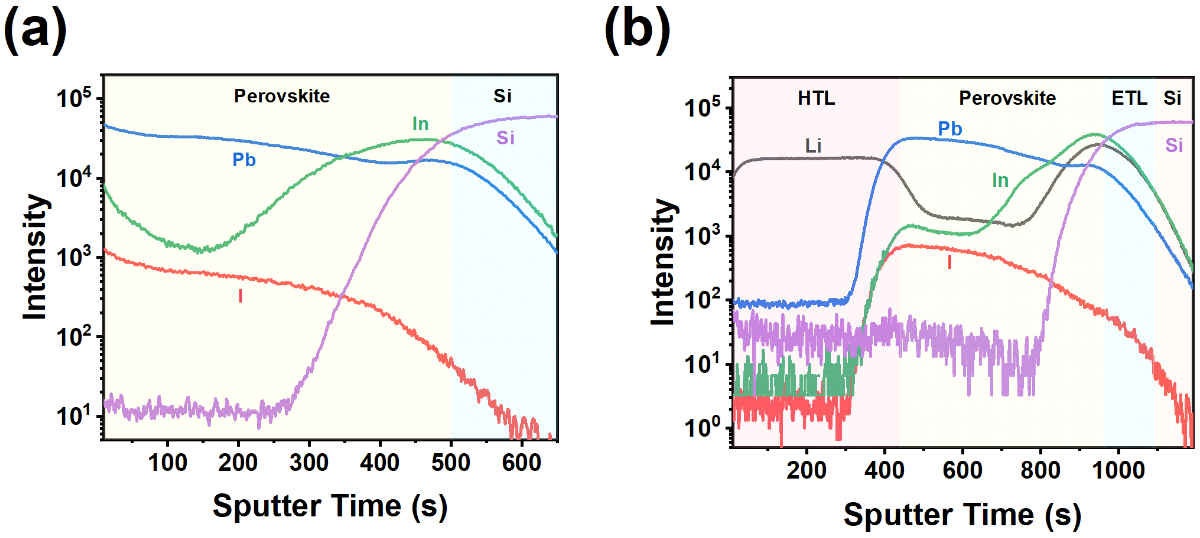

Supplementary Figure 7. The corresponding ToF-SIMS spectra of (a) M:$In_{1.5}$, and (b) M:$In_{1.5}$ devices.

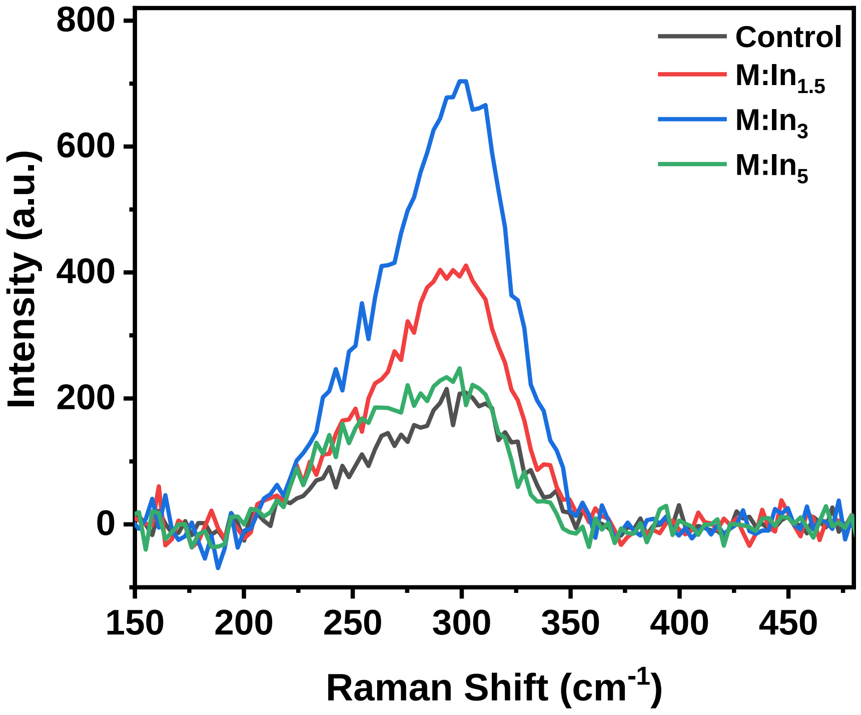

Supplementary Figure 8. Raman spectra of M:In$_x$, where x = 0, 1.5, 3, and 5.

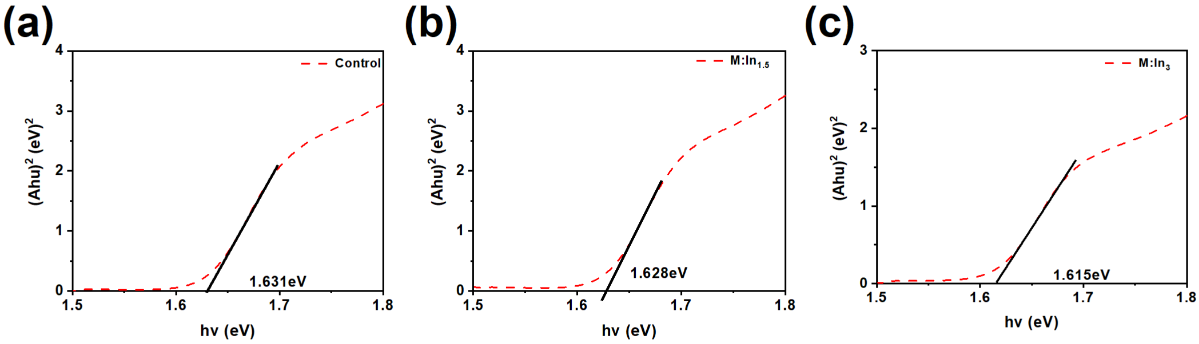

Supplementary Figure 9. Tauc plots of the absorption spectra of (a) control, (b) M:$In_{1.5}$, and (c) M:In_3 thin films.

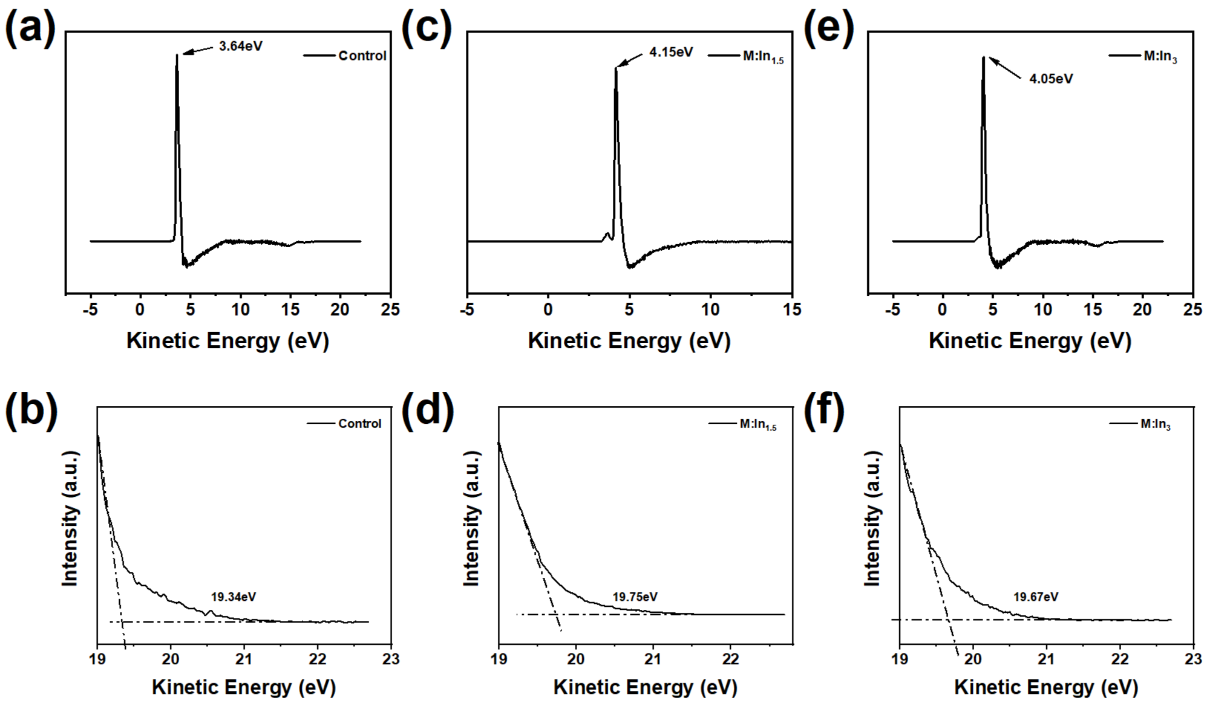

Supplementary Figure 10. First derivative of UPS spectra cutoff region of (a) control, (c) M:$In_{1.5}$, and (e) M:In_3 thin films. VB edge of (b) control, (d) M:$In_{1.5}$, and (f) M:In_3 thin films.

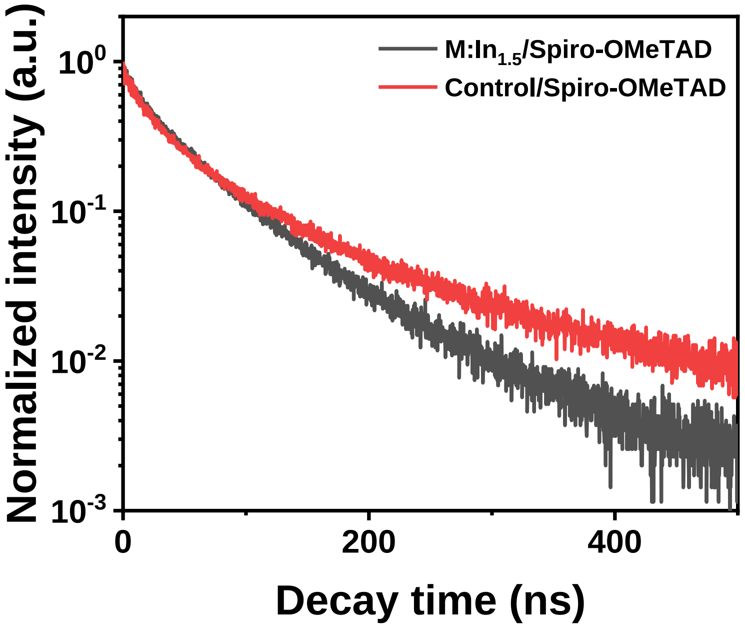

Supplementary Figure 11. TRPL of glass/perovskite/Spiro-OMeTAD.

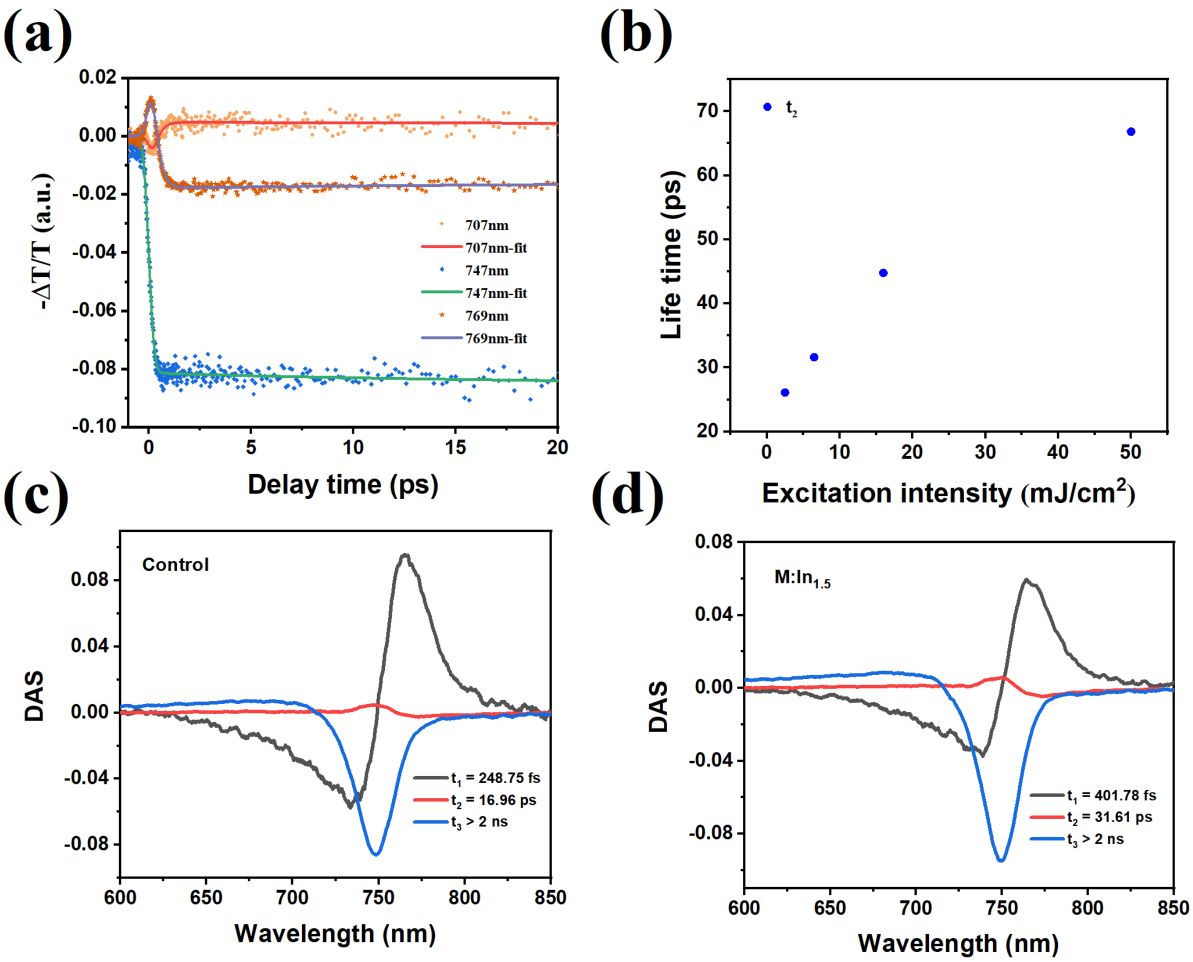

Supplementary Figure 12. (a) Relaxation kinetics of GSB, PIA and hot carrier bleached tail signals within 20 ps of the M:$In_{1.5}$ sample. (b) Dependence of lifetime on excitation intensity in the cooling process of thermalized carriers of the M:$In_{1.5}$ sample. (c, d) decay-associated spectra (DAS) obtained upon global analysis of the TA data.

Global analysis indicates that the PA band is related to the high broadening energy tail of the GSB band, which is consistent with the Fermi–Dirac distribution at high carrier temperatures in the first relaxation process (**Figure S11c and d**). Compared with the control film, M:$ln_{1.5}$ has a longer carrier thermalization time (248.75 vs. 401.78 fs); thus, In^{3+} doping can slow down the rate of the carrier-optical phonon scattering process to reduce the rate of energy loss in the form of heat [2,4–8]. The second relaxation process has a lifetime of tens of picoseconds, and the carriers show the characteristics of the Boltzmann distribution, which is attributed to the cooling process of thermalized carriers[1,7].

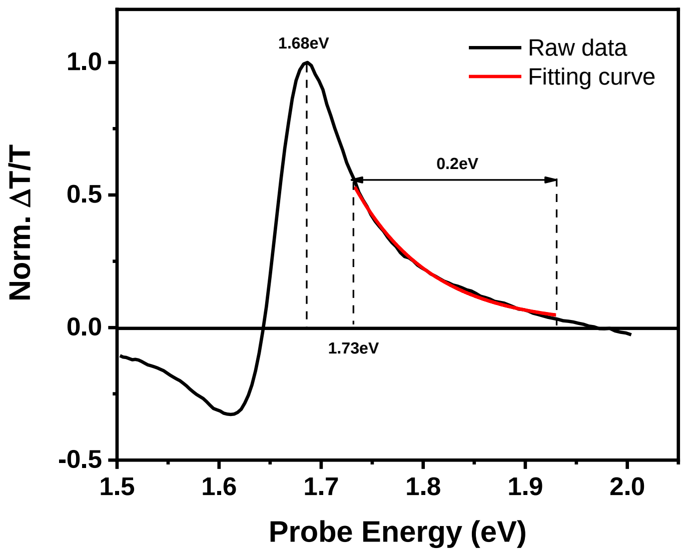

Supplementary Figure 13. High energy tail selection scheme in TA spectra.

To ensure the comparable results of fitted hot-carrier temperatures between different samples. We have adopted the following processing methods, the starting fitting position of high energy tail is 0.05 eV above bleach maximum. And the fitting length has a constant of 0.2 eV from starting position extending to the high energy region. Finally, the Maxwell-Boltzmann equation is used to fitting the selected region to get carrier temperature at different delay times.

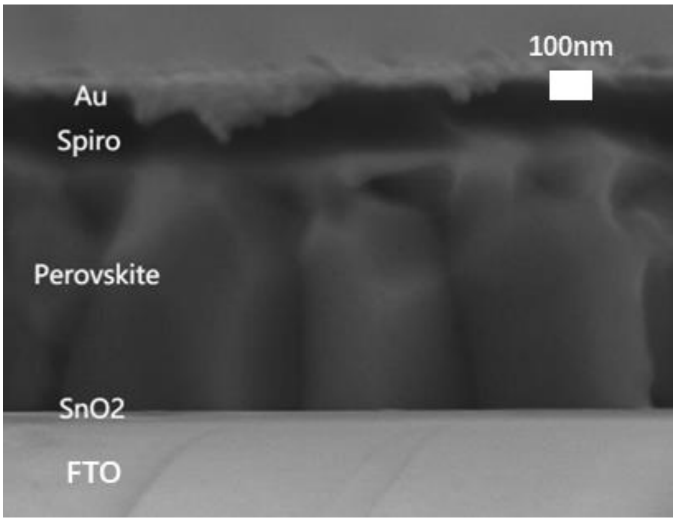

Supplementary Figure 14. Cross-view SEM image of PSCs with the structure of FTO/SnO_2/perovskite/spiro-OMeTAD/Au.

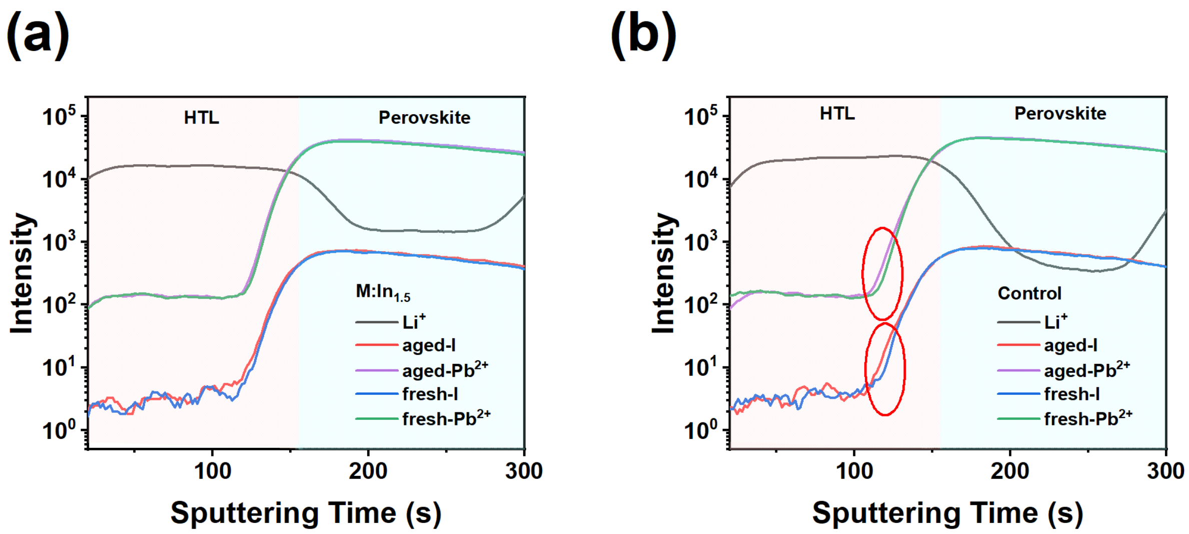

Supplementary Figure 15. ToF-SIMS spectra of fresh and aged devices for (a) M:$In_{1.5}$ sample, (b) control sample.

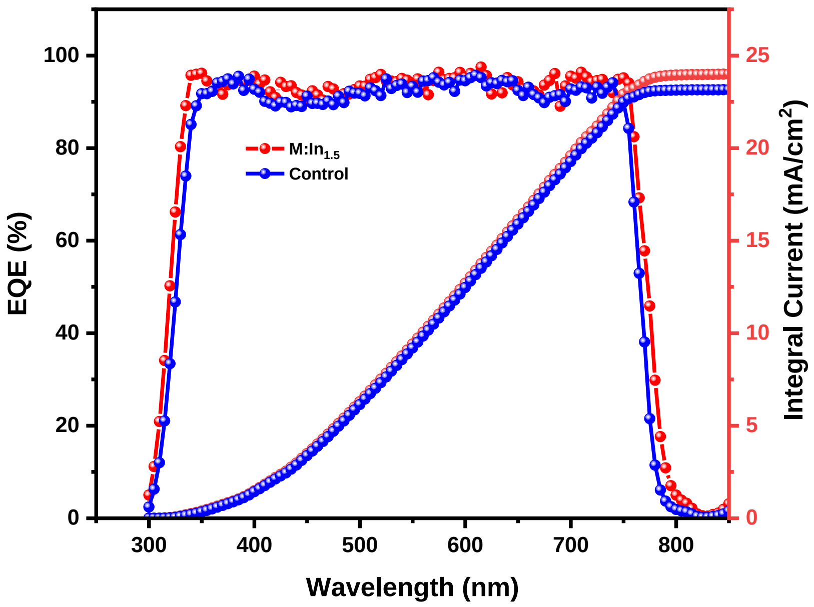

Supplementary Figure 16. The corresponding EQE and integrated current density of the control and $M{:}In_{1.5}$ devices.

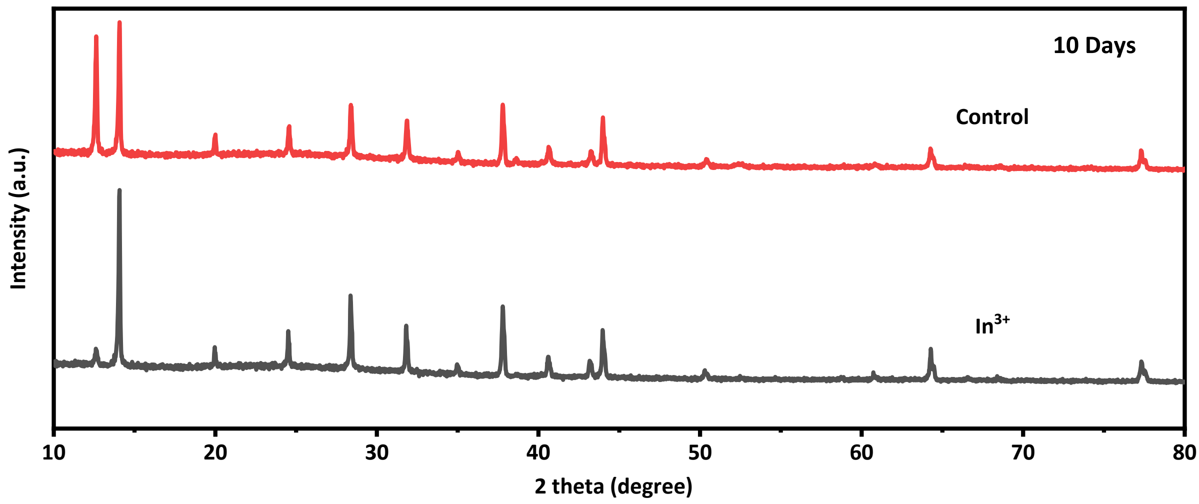

Supplementary Figure 17. XRD patterns of the perovskite thin films w/o In^{3+} incorporation after exposure to the air after 10 days.

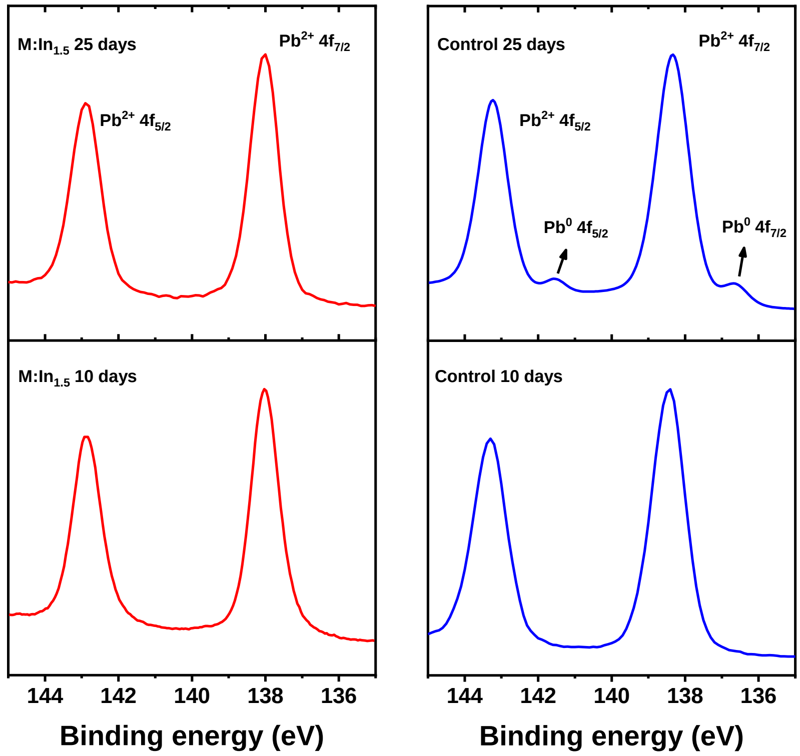

Supplementary Figure 18. XPS spectra of the perovskite thin films w/o In^{3+} incorporation after exposure to the air after 10 and 25 days.

Supplementary Table 1 Carrier lifetimes of the perovskite films.

Sample	τ_1	τ_2
**Control**	4.76	24.3
**M:In$_{1.5}$**	54.0	736
**M:In$_3$**	32.3	394
**SnO$_2$/Control**	6.78	96.3
**SnO$_2$/M:In$_{1.5}$**	6.05	64.7
**Control/Spiro-OMeTAD**	15.4	93.4
**M:In$_{1.5}$/Spiro-OMeTAD**	15.3	65.8

Supplementary Table 2 Average device parameters of the PSCs.

Devices	V_{OC} (V)	J_{SC} (mA cm^{-2})	*FF*	PCE (%)
**Control**	1.10±0.03	23.9±0.7	0.71±0.04	19.0±1.12
**M:In$_{1.5}$**	1.15±0.02	24.4±0.5	0.75±0.04	21.4±0.88
**M:In$_3$**	1.12±0.03	24.0±0.5	0.73±0.03	19.8±0.95
**M:In$_5$**	1.07±0.04	23.5±0.6	0.70±0.05	17.7±1.23

Each value is derived from 30 cells made from 5 separate batches.

References

[1] J. Yang, X. Wen, H. Xia, R. Sheng, Q. Ma, J. Kim, P. Tapping, T. Harada, T. W. Kee, F. Huang, Y.-B. Cheng, M. Green, A. Ho-Baillie, S. Huang, S. Shrestha, R. Patterson, G. Conibeer, *Nat. Commun.* **2017**, *8*, 14120.

[2] H. Kawai, G. Giorgi, A. Marini, K. Yamashita, *Nano Lett.* **2015**, *15*, 3103.

[3] V. Klimov, P. Haring Bolivar, H. Kurz, *Phys. Rev. B* **1995**, *52*, 4728.

[4] J. M. Richter, F. Branchi, F. Valduga de Almeida Camargo, B. Zhao, R. H. Friend, G. Cerullo, F. Deschler, *Nat. Commun.* **2017**, *8*, 376.

[5] M. B. Price, J. Butkus, T. C. Jellicoe, A. Sadhanala, A. Briane, J. E. Halpert, K. Broch, J. M. Hodgkiss, R. H. Friend, F. Deschler, *Nat. Commun.* **2015**, *6*, 8420.

[6] Z. Guo, Y. Wan, M. Yang, J. Snaider, K. Zhu, L. Huang, *Science* **2017**, *356*, 59.

[7] Y. Yang, D. P. Ostrowski, R. M. France, K. Zhu, J. van de Lagemaat, J. M. Luther, M. C. Beard, *Nat. Photonics* **2016**, *10*, 53.

[8] A. Mondal, J. Aneesh, V. Kumar Ravi, R. Sharma, W. J. Mir, M. C. Beard, A. Nag, K. V. Adarsh, *Phys. Rev. B* **2018**, *98*, 115418.
